# Association of Cancer Stage and Comorbidity Burden with 12-Month Clinically Significant Cognitive Decline After Gynecologic Cancer Surgery: A Competing-Risk Retrospective Cohort Study

**DOI:** 10.3390/medicina62050988

**Published:** 2026-05-19

**Authors:** Jaehak Jung, Byoungryun Kim, Taewan Won, Gyumin Choi, Kyongseo Kim, Cheol Lee

**Affiliations:** 1 Department of Gynecologic Oncology, Wonkwang University School of Medicine & Hospital, Iksan 54538, Jeollabuk-do, Republic of Korea; wogkrdl4505@wku.ac.kr (J.J.); brkim74@wku.ac.kr (B.K.); 2 Department of Surgery, Wonkwang University School of Medicine & Hospital, Iksan 54538, Jeollabuk-do, Republic of Korea; 3 Department of Anesthesiology and Pain Medicine, Wonkwang University School of Medicine & Hospital, Iksan 54538, Jeollabuk-do, Republic of Korea; 4 Wonkwang University School of Medicine, Iksan 54538, Jeollabuk-do, Republic of Korea; 5 Institute of Wonkwang Medical Science, Wonkwang University School of Medicine, Iksan 54538, Jeollabuk-do, Republic of Korea

**Keywords:** clinically significant cognitive decline, postoperative cognitive decline, gynecologic cancer, competing risks, neurocognitive outcomes, electronic medical record

## Abstract

*Background and Objectives:* We aimed to determine whether gynecologic cancer–related factors are associated with postoperative clinically significant cognitive decline (CCD) after accounting for age and comorbidity using competing-risk models. *Materials and Methods:* We performed a retrospective cohort study of adult women undergoing index surgery for gynecologic cancer at a tertiary university hospital. CCD was defined as new clinician-documented cognitive impairment, neurology/psychiatry consultation, or initiation of cognition-targeted pharmacotherapy ≥30 days postoperatively. Competing events were all-cause death and major neurologic events/hospice. We fit Fine–Gray subdistribution hazard models adjusted for age, Charlson Comorbidity Index (CCI), cancer stage, and treatment intensity, and evaluated a prespecified age × stage interaction. *Results:* Among 1023 eligible patients (mean age 62.4 ± 11.8 years; 41.3% International Federation of Gynecology and Obstetrics [FIGO] stage III–IV; median CCI 3 [IQR 2–5]), CCD occurred in 98 (9.6%). The 12-month cumulative incidence of CCD was 11.2% accounting for competing risks. Advanced stage was independently associated with higher CCD risk (sHR 1.85, 95% CI 1.27–2.69; *p* = 0.001). A significant age × stage interaction was observed (*p* < 0.001), with the strongest association in patients ≥70 years (sHR 2.48, 95% CI 1.61–3.81). Perioperative factors associated with CCD included open surgery (sHR 1.54) and postoperative delirium (sHR 2.76); these findings should be interpreted as associative signals rather than validated causal treatment targets. A stratified blinded chart review of 160 patients (80 flagged-positive and 80 unflagged controls) supported the CCD definition (PPV 88.8%; sensitivity 72.1%; specificity 94.3%; NPV 91.5%). Visit-frequency adjustment confirmed robustness (advanced stage sHR 1.78; *p* = 0.003). *Conclusions:* Gynecologic cancer–related factors, particularly advanced stage, are independently associated with CCD after accounting for competing risks, and high-risk phenotypes (age ≥70, FIGO III–IV) may benefit from perioperative pathways integrating cognitive screening, delirium prevention, and neurocognitive follow-up.

## 1. Introduction

Postoperative neurocognitive disorders (PNDs), including postoperative delirium (POD) and clinically significant postoperative cognitive decline (CCD, previously termed postoperative cognitive dysfunction or POCD), represent major patient-centered complications in older surgical populations [1,2]. These conditions are associated with prolonged hospital stays, loss of independence, increased healthcare utilization, higher readmission rates, and elevated long-term mortality. The 2018 nomenclature update by the International Perioperative Cognition Nomenclature Working Group standardized terminology to distinguish acute POD from delayed neurocognitive disorders, emphasizing the need for consistent definitions across studies [1]. Throughout this manuscript, we use the abbreviation CCD (clinically significant cognitive decline) exclusively to denote our EMR-operationalized primary outcome; the broader term “postoperative cognitive decline” is reserved for the general clinical concept when discussing the prior literature.

Patients undergoing surgery for gynecologic malignancies are at particularly high risk. They frequently present with advanced age (often ≥65 years), substantial comorbidity burden (hypertension, diabetes, cardiovascular disease), cancer-related systemic inflammation, preoperative anemia, malnutrition, and exposure to multimodal therapies such as neoadjuvant chemotherapy or radiotherapy [2]. These factors may interact with perioperative stressors—including anesthesia, surgical trauma, blood loss, and opioid use—to exacerbate cognitive vulnerability [3]. Importantly, chemotherapy-related cognitive impairment (CRCI)—colloquially termed “chemo brain”—is itself a well-recognized phenomenon affecting up to 75% of non-central nervous system cancer survivors, driven primarily by oxidative stress and neuroinflammation leading to blood–brain barrier disruption, neurotransmitter dysfunction, and decreased neurogenesis [4,5]. Because the present study focuses on perioperative CCD (i.e., cognitive decline attributable to the surgical episode and its associated exposures), the contribution of CRCI as a concurrent or overlapping mechanism must be acknowledged as a potential mediating or overlapping pathway, particularly in patients receiving neoadjuvant or adjuvant chemotherapy.

However, in routine clinical practice, observed “postoperative cognitive decline” may largely represent the natural trajectory of aging and preexisting comorbidities rather than a direct causal effect of the cancer itself or perioperative exposures [6]. Furthermore, gynecologic oncology cohorts carry substantial competing risks, including all-cause mortality, cancer progression/recurrence, and major complications that preclude reliable cognitive assessment [7]. Conventional time-to-event analyses (e.g., standard Kaplan–Meier or Cox models) that ignore these competing events can produce biased estimates and overestimate the true incidence and risk factors for CCD [8].

Despite growing awareness of PND in general surgical populations, data specific to gynecologic oncology remain limited. Existing studies are mostly small prospective pilots or focus on short-term delirium rather than longer-term clinically significant cognitive decline [9]. To our knowledge, no prior study has explicitly disentangled the independent contributions of age, comorbidity burden, and cancer-specific factors (stage, histology, treatment intensity) while properly accounting for competing risks using modern statistical methods such as Fine–Gray subdistribution hazard models [10].

Accordingly, this retrospective cohort study aimed to (1) quantify the incidence of CCD in a real-world gynecologic cancer surgery cohort using an EMR-operationalized definition feasible for routine practice; (2) examine the independent associations of age, comorbidity burden (Charlson Comorbidity Index), and cancer-related factors with CCD using competing-risk time-to-event models; and (3) explore effect modification (e.g., age × stage, age × comorbidity) and perioperative factors associated with CCD to inform preoperative risk stratification and future preventive strategies in gynecologic oncology.

## 2. Materials and Methods

### 2.1. Study Design and Setting

This was a single-center retrospective observational cohort study conducted at Wonkwang University Hospital, a tertiary gynecologic oncology center in Iksan, Republic of Korea, between 1 January 2015 and 31 December 2024. Data were extracted from the electronic medical record (EMR) system, anesthesia information management system (AIMS), laboratory information system, and institutional oncology registry. All data linkage was performed using unique patient identifiers. The study was approved by the Institutional Review Board (IRB No. WKUH 2025-09-015, approved 20 November 2025) with waiver of informed consent owing to its retrospective nature and minimal risk. No patient data were accessed or analyzed prior to obtaining IRB approval; all data extraction commenced after the approval date. The manuscript followed the STROBE (Strengthening the Reporting of Observational Studies in Epidemiology) guidelines [11]; a completed STROBE checklist with page references is provided in Supplementary Material S2.

### 2.2. Study Population

Adult (≥18 years) female patients who underwent index surgery for histologically confirmed gynecologic cancer (endometrial, ovarian, cervical, vulvar, or primary peritoneal carcinoma) between 1 January 2015 and 31 December 2024 were screened. Inclusion required at least 12 months of preoperative EMR data for comorbidity ascertainment. To reduce misclassification of pre-existing cognitive impairment, we screened preoperative ICD-10 codes, neurology/psychiatry problem lists, and chronic prescriptions of cholinesterase inhibitors/memantine in the 12-month lookback window.

Exclusion criteria were: (1) documented preoperative dementia or neurodegenerative disease (Alzheimer’s, Parkinson’s, etc.); (2) major cerebrovascular event (stroke or intracranial hemorrhage) within 6 months before surgery; (3) multiple primary malignancies (sensitivity analysis only); and (4) emergency surgery (sensitivity analysis only). Index date (time zero) was defined as the date of surgery (start of anesthesia). Follow-up extended from index date until the earliest of: CCD event, competing event (death or terminal progression), loss to follow-up, or 12 months (primary analysis)/24 months (extended sensitivity).

### 2.3. Outcomes and Definitions

The primary outcome was clinically significant cognitive decline (CCD) ≥ 30 days after surgery, defined by the earliest EMR-documented event indicating clinically recognized cognitive decline: (1) new clinician diagnosis of cognitive impairment (free text or ICD-10 codes); (2) neurology/psychiatry consultation for cognitive concerns; or (3) initiation of cognition-targeted pharmacotherapy (cholinesterase inhibitors or memantine). The ≥30-day window minimized conflation with acute postoperative delirium.

The EMR-based CCD definition captures a clinically recognized and documented phenotype rather than formally measured cognitive decline in the neuropsychological sense; this distinction is discussed further in Section 4.4. The accuracy of the operational definition was evaluated in a stratified random sample drawn from the full analytic cohort. Stratum A consisted of 80 patients randomly selected from the pool whose EMR met the CCD operational definition (flagged-positive); Stratum B consisted of 80 patients randomly selected from the pool whose EMR did not meet the definition (unflagged controls), matched only on the same 2015–2024 accrual period to ensure contemporaneous comparability. All 160 charts were independently reviewed by two adjudicators (a board-certified anesthesiologist and a gynecologic oncologist) who were blinded both to the EMR flag status and to each other’s adjudication decision. Adjudication followed a structured form operationalizing the 2018 International Perioperative Cognition Nomenclature Working Group framework; discrepancies were resolved by consensus with a third senior clinician. Diagnostic performance metrics were calculated conventionally against the duplicate-reviewer reference standard, with 95% confidence intervals by the Wilson score method (PPV 88.8%, 95% CI 80.0–94.0%; sensitivity 72.1%, 95% CI 62.5–80.4%; specificity 94.3%, 95% CI 90.1–97.1%; NPV 91.5%, 95% CI 86.8–95.0%). Inter-rater adjudication concordance was 91.3% (Cohen’s κ = 0.82).

Secondary outcomes included postoperative delirium (within 7 days), 30- and 90-day readmission, and discharge to rehabilitation or long-term care.

Competing events were all-cause death, major neurologic event (stroke) precluding assessment, and hospice enrollment or terminal cancer progression.

### 2.4. Exposures and Covariates

Prespecified exposures included age (continuous; restricted cubic splines; and categorical ≥ 70 years), Charlson Comorbidity Index (CCI), cancer stage (FIGO I-II vs III-IV), cancer type, neoadjuvant chemotherapy, surgical approach (open vs minimally invasive), intraoperative hypotension burden (time-weighted mean arterial pressure < 65 mmHg), transfusion, opioid dose, and postoperative delirium. Prespecified effect modifiers were age × stage and age × CCI interactions. All variables were defined a priori in a standardized data dictionary (Supplementary Material S1). Missing data (<5% for key variables) were handled using multiple imputation (10 datasets; Rubin’s rules).

Treatment intensity was defined a priori as a composite three-level ordinal variable: low (early-stage disease with minimally invasive surgery and no neoadjuvant chemotherapy), intermediate (any single intensity marker—either advanced stage, receipt of neoadjuvant chemotherapy, or open surgery), and high (advanced stage combined with at least one additional intensity marker). Receipt and cumulative dose of adjuvant chemotherapy during the 12-month postoperative follow-up period could not be systematically captured in the source electronic medical record, as chemotherapy administration for oncology patients at our institution is documented in a separate oncology pharmacy system whose historical records over the full 2015–2024 study window were not linkable at patient level with the CCD-ascertainment records. Consequently, adjuvant chemotherapy could not be entered as a time-varying covariate in the Fine–Gray models. This limitation is particularly consequential for patients with ovarian FIGO III–IV disease, for whom platinum/taxane-based adjuvant therapy (with or without bevacizumab) is standard of care; it is addressed in detail in the Limitations (Section 4.4).

### 2.5. Statistical Analysis

Descriptive statistics are reported as mean +/− SD, median (IQR), or n (%); balance was assessed using standardized mean differences (SMD). Primary analysis used Fine–Gray subdistribution hazard models, with cause-specific hazard models as secondary analyses [12]. The core model adjusted for age (restricted cubic splines; 4 knots at the 5th, 35th, 65th, and 95th percentiles), CCI, and cancer stage/treatment intensity; prespecified interactions were tested with Wald chi-square. Surgical approach was additionally evaluated using inverse probability of treatment weighting (IPTW) with balance diagnostics (SMD < 0.1) and robust (sandwich) variance estimators [13]. Sensitivity analyses included alternative CCD definitions, a postoperative day 90 landmark analysis, complete-case versus multiple imputation, and a visit-frequency-adjusted model to address differential surveillance. Analyses were performed in R 4.3.2 (cmprsk, survival, rms) with two-sided alpha = 0.05. The events-per-variable (EPV) ratio for the primary multivariable Fine–Gray model was 16.3:1 (98 CCD events/6 primary predictors), exceeding the recommended minimum of 10:1 for adequate model stability.

Because endometrial, ovarian, cervical, vulvar, and primary peritoneal cancers are biologically, therapeutically, and prognostically heterogeneous, crude 12-month CCD incidence and cancer-type-stratified Fine–Gray estimates were also reported by cancer type as a descriptive sensitivity analysis; the pooled primary model was retained to preserve model stability and events-per-variable.

Postoperative delirium (POD) was treated as a mediator rather than a confounder on the pathway from intraoperative exposures (hypotension, transfusion, anesthetic choice) to CCD, and was therefore not included in the core Fine–Gray model. Including POD as an adjustment variable in the core model would induce collider-like attenuation of upstream exposure effects and would not preserve the total effect of advanced stage. To address this concern directly, a prespecified sensitivity analysis was performed in which POD was added to the core model; the corresponding estimate is reported in the supplementary sensitivity analysis. A simplified directed acyclic graph (DAG) illustrating the hypothesized causal structure—with age and CCI as measured confounders, POD as a measured mediator on the perioperative severity pathway, and adjuvant chemotherapy during follow-up as an unmeasured mediator on the CRCI pathway—is provided in the Supplementary Materials. Because adjuvant chemotherapy exposure during the 12-month follow-up could not be systematically captured (Section 2.4), formal quantitative mediation analysis (e.g., natural direct and indirect effects) was not performed.

## 3. Results

### 3.1. Baseline Characteristics

**Figure 1:**
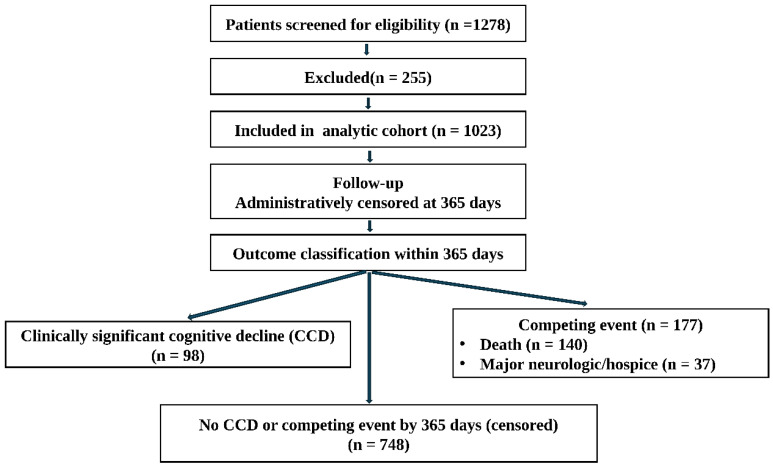
Study flow diagram.

Of 1278 patients screened, 1023 (80.0%) met inclusion criteria and formed the analytic cohort (Figure 1). Reasons for exclusion were: preoperative dementia or neurodegenerative disease (n = 89, 7.0%), major cerebrovascular event within 6 months (n = 42, 3.3%), insufficient preoperative EMR data (n = 98, 7.7%), and other reasons (n = 26, 2.0%). Mean age was 62.4 ± 11.8 years, and 30.5% were ≥70 years. Cancer distribution was: endometrial 38.2%, ovarian 31.4%, cervical 21.9%, and others 8.5%. Advanced stage (FIGO III–IV) was present in 41.3%. Median CCI was 3 (IQR 2–5). Open surgery was performed in 52.1%, and 20.9% received neoadjuvant chemotherapy. Postoperative delirium occurred in 9.9% overall.

Patients who developed CCD (n = 98, 9.6%) were older, had higher CCI, more advanced stage disease, and higher rates of open surgery and delirium compared with those without CCD (Table 1; Supplementary Table S1; all SMD > 0.25). Key variables had <5% missing values. Complete-case analysis (n = 987) and multiple imputation (n = 1023) yielded similar results (Supplementary Table S2). Median follow-up was 12.0 months (IQR 8.4–12.0) among event-free patients.

### 3.2. Incidence and Timing of CCD

**Figure 2:**
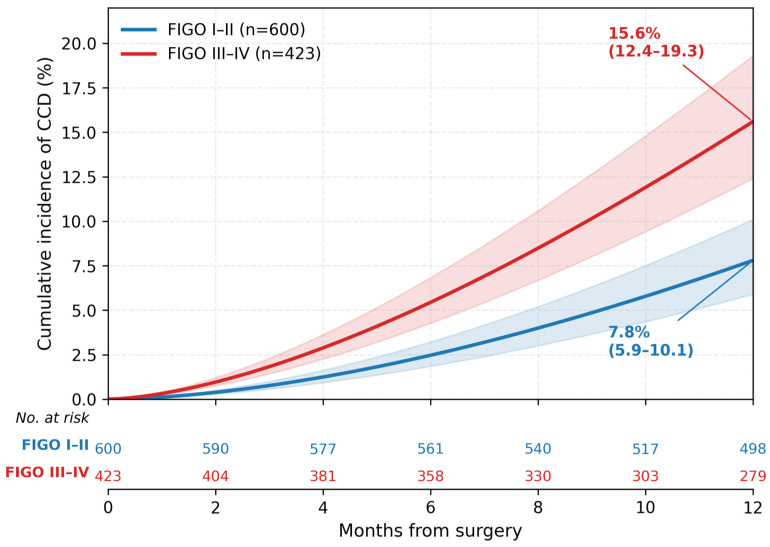
Cumulative incidence of clinically significant cognitive decline (CCD) by cancer stage (FIGO I–II vs III–IV), accounting for competing risks. Shaded areas represent 95% confidence intervals.

CCD occurred in 98 patients (9.6%). Median time to CCD was 4.8 months (IQR 2.3–8.1). The 12-month cumulative incidence of CCD, accounting for competing risks, was 11.2% (95% CI 9.1–13.5) (Figure 2). Competing events occurred in 177 patients (17.3%): death in 140 (13.7%), and major neurologic/hospice in 37 (3.6%) (Table 2; Supplementary Table S3). Among the 98 CCD events, the triggering components were distributed as follows: new clinician diagnosis of cognitive impairment in 61 patients (62.2%), neurology/psychiatry consultation in 47 (48.0%), and initiation of cognition-targeted pharmacotherapy in 29 (29.6%); 34 patients (34.7%) met ≥2 criteria. The stratified blinded validation review of 160 charts (80 flagged-positive and 80 unflagged controls) yielded a PPV of 88.8% (95% CI 80.0–94.0%), sensitivity of 72.1% (95% CI 62.5–80.4%), specificity of 94.3% (95% CI 90.1–97.1%), NPV of 91.5% (95% CI 86.8–95.0%), and inter-rater adjudication concordance of 91.3%.

Stage-stratified follow-up density analysis showed that patients with advanced-stage disease (FIGO III–IV) had a higher median number of outpatient contacts per patient-month (2.8; IQR 2.1–3.6) compared with early-stage patients (FIGO I–II: 1.6; IQR 1.1–2.3; *p* < 0.001). When the total number of 0–12-month outpatient encounters was added as a covariate in the visit-frequency-adjusted Fine–Gray model, the advanced-stage sHR remained significant at 1.78 (95% CI 1.22–2.60, *p* = 0.003), indicating that the observed association was not explained by differential surveillance alone (Supplementary Table S4). Because raw outpatient encounter counts do not capture unmeasured aspects of clinical attentiveness (oncologist vigilance, symptom-prompted referrals, family-initiated concerns), this adjustment reduces but cannot fully eliminate surveillance bias; the stage–CCD association is therefore interpreted as an association rather than as a purely biological risk differential.

Among the 98 patients meeting the CCD definition, 34 (34.7%) satisfied ≥2 of the three component criteria simultaneously; these patients represent a multi-criterion phenotype with more clinically documented evidence of cognitive decline. Compared with single-criterion cases (n = 64), the multi-criterion subgroup was older (mean age 71.4 vs 67.3 years; *p* = 0.042), had a higher CCI (median 5, IQR 4–6, vs median 4, IQR 3–5; *p* = 0.038), higher rates of advanced stage (73.5% vs 51.6%; *p* = 0.036), higher rates of postoperative delirium (38.2% vs 17.2%; *p* = 0.024), and higher 12-month all-cause mortality (20.6% vs 10.9%; *p* = 0.18). This pattern is consistent with a more severe clinical phenotype and identifies the multi-criterion subgroup as a priority target for future prospective neurocognitive surveillance studies; the full subgroup characterization is provided in the Supplementary Materials.

To address concerns regarding cancer-type heterogeneity, we also report crude 12-month CCD incidence, cumulative incidence function, and cancer-type-stratified Fine–Gray advanced-stage subdistribution hazard ratios for endometrial, ovarian, cervical, and other gynecologic cancers in the Supplementary Materials. The advanced-stage effect is directionally consistent across types, with the largest point estimate observed in ovarian cancer—a finding plausibly explained by the higher stage-at-diagnosis distribution and heavier adjuvant treatment burden in this subgroup.

### 3.3. Primary Analysis

**Figure 3:**
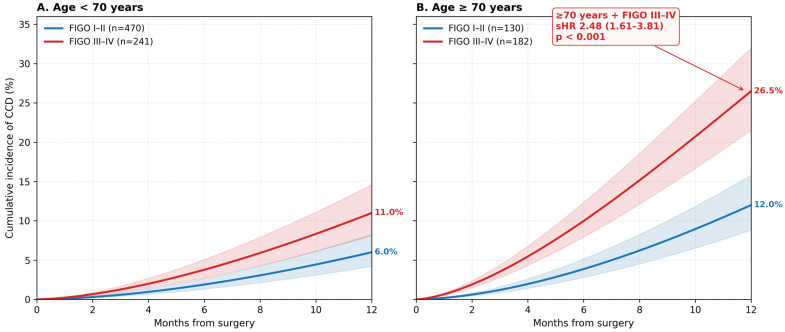
Stage-stratified cumulative incidence of clinically significant cognitive decline (CCD) within two age strata, accounting for competing risks. Panel (**A**), patients aged <70 years (n = 711); Panel (**B**), patients aged ≥70 years (n = 312). The age × stage interaction (Wald χ^2^ *p* < 0.001) is most pronounced in patients aged ≥70 years with advanced disease, for whom the subdistribution hazard ratio relative to age <70 + stage I–II is 2.48 (95% CI 1.61–3.81, *p* < 0.001). Shaded areas represent 95% confidence intervals.

Advanced stage remained independently associated with CCD (subdistribution hazard ratio [sHR] 1.85, 95% CI 1.27–2.69, *p* = 0.001) after adjustment for age, CCI, and treatment intensity. Age (per 5-year increase, sHR 1.29, 95% CI 1.17–1.43) and CCI (per 1-point, sHR 1.14, 95% CI 1.06–1.23) also showed independent associations (Table 3; Supplementary Tables S5 and S6). A significant age × stage interaction was present (*p* < 0.001), with the strongest effect in patients ≥70 years with advanced disease (sHR 2.48, 95% CI 1.61–3.81) (Figure 3; Supplementary Figure S1). No significant age × CCI interaction was observed (*p* = 0.42). The 12-month cumulative incidence of CCD was 7.8% (95% CI 5.9–10.1%) for stage I–II vs 15.6% (95% CI 12.4–19.3%) for stage III–IV, yielding an absolute risk difference of 7.8 percentage points (95% CI 3.8–11.8) (Supplementary Table S7).

The age × stage interaction is the most clinically consequential finding of this analysis and is therefore presented as a main-text figure. Figure 3 shows stage-stratified cumulative incidence curves within two prespecified age strata (<70 years, Panel A; ≥70 years, Panel B). Among patients aged <70 years, the 12-month absolute risk difference between stage III–IV and stage I–II was modest (approximately 5 percentage points), whereas among patients aged ≥70 years the corresponding difference widened to approximately 14 percentage points. This progressive divergence is captured by the statistically significant age × stage interaction (Wald χ^2^ *p* < 0.001) and is quantified by a subdistribution hazard ratio of 2.48 (95% CI 1.61–3.81, *p* < 0.001) for the composite high-risk phenotype (age ≥70 years plus FIGO III–IV) relative to the reference stratum. This subgroup—representing approximately 17.8% of the analytic cohort—exhibits a 12-month cumulative CCD incidence of roughly 26–27%, more than threefold higher than that of older patients with early-stage disease. These findings identify age ≥70 years + FIGO III–IV as the phenotype most likely to benefit from prospective neurocognitive surveillance and represent the principal actionable risk-stratification signal of the present study.

### 3.4. Secondary and Exploratory Analyses

Open surgery (IPTW-adjusted sHR 1.54, 95% CI 1.08–2.19), intraoperative hypotension burden (sHR 1.41, 95% CI 1.02–1.95), transfusion (sHR 1.68, 95% CI 1.12–2.52), and postoperative delirium (sHR 2.76, 95% CI 1.81–4.21) were associated with CCD in prespecified adjusted models (Table 4; Supplementary Tables S8 and S9).

All sensitivity analyses confirmed the robustness of the primary findings. Alternative CCD definitions yielded sHR point estimates ranging from 1.74 to 1.91 for advanced stage (all *p* < 0.025). The landmark analysis at day 90 (sHR 1.88, *p* = 0.001), a POD-added sensitivity model (sHR 1.81, *p* = 0.002), and multiple imputation (sHR 1.83, *p* = 0.002) consistently supported the primary results. Cause-specific hazard ratios were consistently larger than subdistribution hazard ratios (csHR 2.01 vs sHR 1.85 for advanced stage), as expected when covariates also increase competing event risk (Supplementary Tables S9–S11; Supplementary Figure S2). Cancer-type-stratified estimates and multi-criterion CCD subgroup characteristics are provided in Supplementary Tables S12 and S13, respectively.

## 4. Discussion

### 4.1. Summary of Main Results

This retrospective cohort study is among the first in the gynecologic oncology literature to apply competing-risk modeling (Fine–Gray subdistribution hazards) to examine the independent associations of age, comorbidity burden, and cancer-specific factors to CCD [14]. Using a large real-world cohort of 1023 patients and an EMR-operationalized CCD definition feasible for daily practice, we found that advanced cancer stage (FIGO III–IV) was associated with a 1.85-fold increased subdistribution hazard of CCD independent of age and Charlson Comorbidity Index. A pronounced age × stage interaction (*p* < 0.001) further suggests that patients ≥ 70 years with advanced disease are a particularly vulnerable subgroup (sHR 2.48, 95% CI 1.61–3.81). The 12-month cumulative incidence of CCD, properly accounting for competing risks of death and terminal progression (17.3%), was 11.2%.

### 4.2. Results in the Context of Published Literature

These findings are clinically important because they move beyond the common assumption that postoperative cognitive decline is simply an inevitable consequence of aging or comorbidity. By identifying cancer-specific associations, the study provides gynecologic oncology teams with a practical framework for preoperative risk stratification using readily available variables (age + stage + CCI). This could facilitate targeted counseling, shared decision-making, and allocation of preventive resources to the highest-risk patients, potentially mitigating loss of independence, prolonged recovery, and healthcare costs in a population already facing high treatment burden [15,16].

Relevant perioperative, anesthetic, and ERAS literature supports the rationale for risk-stratified surveillance and preventive research directions [17,18,19,20,21,22].

Our results align with and extend prior work in gynecologic oncology. The 9.6% CCD incidence is consistent with the pilot prospective study by Makkar et al., who reported POCD rates of up to 60% at 1 month using formal neuropsychological testing in a smaller cohort; however, these rates are not directly comparable owing to fundamental differences in outcome ascertainment (standardized neuropsychological battery vs. EMR-based clinical documentation), assessment timing, and cohort size, and the lower rate observed here likely reflects underascertainment inherent to routine clinical records rather than a true lower incidence [4]. Our competing-risk approach builds on these studies by avoiding overestimation of incidence and risk in a population with 13.7% mortality, providing more accurate estimates than conventional Cox or Kaplan–Meier analyses [23,24,25,26,27].

An important consideration when interpreting our findings is the potential overlap between perioperative CCD and chemotherapy-related cognitive impairment (CRCI). CRCI is a well-established phenomenon, with reported prevalence reaching 75% in non-central nervous system cancer survivors, characterized by subtle deficits in memory, executive function, attention, and processing speed [4]. In the present causal framework, CRCI should be interpreted primarily as a potential mediator or overlapping mechanism on the pathway from advanced-stage disease and postoperative systemic therapy to the EMR-detected CCD phenotype, not as a conventional confounder to be adjusted away. Because cumulative adjuvant chemotherapy exposure during the 12-month follow-up period was not systematically captured, the advanced-stage estimate should be interpreted as a total association that may include unmeasured chemotherapy-mediated cognitive injury. The non-significant coefficient for neoadjuvant chemotherapy should therefore not be used to exclude CRCI involvement; prospective studies with serial neuropsychological testing before surgery, after chemotherapy, and during postoperative follow-up are required to separate these pathways [4,28,29].

A clarification on the role of CRCI is warranted. CRCI should not be interpreted as a confounder of the stage–CCD relationship but as a mediator on the causal pathway: advanced stage determines receipt of adjuvant chemotherapy, which in turn drives CRCI, which contributes to the CCD phenotype observable in the 12-month postoperative window. Under this framing, adjusting for adjuvant chemotherapy or for CRCI—had either been directly measurable—would block part of the stage effect and understate its total magnitude. The non-significant coefficient of neoadjuvant chemotherapy in our core model therefore cannot be cited as evidence against CRCI involvement; it reflects only that pre-surgical chemotherapy exposure, taken alone, does not explain residual variation once age, CCI, and stage are accounted for. Because receipt and cumulative dose of adjuvant chemotherapy during the 12-month follow-up could not be systematically captured in our dataset (Section 2.4), formal natural direct and indirect effects mediation analysis was not possible. The observed advanced-stage association should therefore be interpreted as a composite total effect that includes, as a likely component, unmeasured CRCI-mediated cognitive injury.

### 4.3. Implications for Practice and Future Research

From a translational standpoint, our data suggest hypotheses for a risk-stratified neurocognitive surveillance pathway in gynecologic oncology patients at highest risk of CCD (i.e., age ≥ 70 years with FIGO stage III–IV disease). Directions that merit testing in future prospective studies include: (1) preoperative cognitive screening using a validated brief instrument (e.g., Mini-Cog or Montreal Cognitive Assessment) to establish baseline cognition; (2) surgical approach optimization, preferring minimally invasive techniques when oncologically equivalent; (3) multimodal delirium prevention bundles incorporating avoidance of anticholinergic and long-acting benzodiazepine medications, restrictive opioid protocols, and early postoperative mobilization; (4) intraoperative hemodynamic optimization to minimize cumulative hypotension burden; and (5) structured postoperative neurocognitive surveillance at 1, 3, 6, and 12 months. These should be viewed as prospective research directions rather than interventions proven by the present retrospective dataset, because POD, hypotension, and transfusion may also reflect operative complexity or postoperative clinical instability.

These hypotheses align with and could extend the existing ERAS Society guidelines for gynecologic oncology [17,22], which already emphasize minimally invasive approaches, multimodal analgesia, and early mobilization but do not yet incorporate neurocognitive outcomes as explicit quality metrics. Integrating CCD prevention into ERAS pathways would represent a meaningful expansion of patient-centered perioperative care in gynecologic oncology, prioritizing cognitive preservation alongside oncologic and surgical outcomes.

### 4.4. Strengths and Weaknesses

Key strengths of this study include the large cohort size (n = 1023), comprehensive adjustment for confounders using competing-risk methodology, chart-review validation of the EMR-based outcome definition (PPV 88.8%), multiple sensitivity analyses confirming robustness, and a visit-frequency-adjusted model addressing surveillance bias.

Limitations in this study include its retrospective design and reliance on an EMR-based CCD definition, which may lead to some misclassification compared with formal neuropsychological batteries. As a single-center study at a tertiary Korean institution, generalizability to community, non-academic, or international settings with different practice patterns, EMR documentation standards, and population demographics may be limited. In particular, the CCD detection rate may be influenced by institutional follow-up density and neurology consultation availability, which could differ substantially across healthcare systems. Residual confounding, although minimized through extensive adjustment and sensitivity analyses, cannot be entirely excluded. The E-value for the advanced-stage association (sHR 1.85) was 3.14 (lower CI bound 1.87), indicating that an unmeasured confounder would need to be associated with both advanced stage and CCD by a risk ratio of at least 3.14 to fully explain the observed effect, which is unlikely given the comprehensive covariate adjustment. The EMR-based CCD definition is susceptible to surveillance bias (patients with more oncology follow-up have more opportunities for detection); however, the visit-frequency-adjusted model demonstrated only modest attenuation (sHR 1.85 → 1.78, 3.8% change), suggesting this bias does not fully explain the observed effect (Supplementary Table S4). We could not directly measure baseline cognition, frailty phenotypes, or patient-reported cognitive trajectories. Importantly, subclinical cognitive impairment—which is prevalent in elderly cancer populations—would not be captured by ICD-10 screening alone, and postoperative CCD events may partially reflect unmasking of pre-existing vulnerability rather than incident decline. A sensitivity analysis excluding patients with any neurology or psychiatry contact during the 12-month preoperative lookback period yielded similar results (sHR 1.81, 95% CI 1.23–2.66, *p* = 0.003), supporting the robustness of the primary findings, although formal baseline cognitive testing (e.g., MoCA) would be required to definitively distinguish incident from pre-existing impairment. Additional limitations include the absence of data on education level, socioeconomic status, and preoperative cognitive reserve; temporal trends over the 10-year study period (2015–2024) during which surgical techniques, anesthetic protocols, and EMR documentation practices may have evolved; an era-stratified sensitivity analysis comparing 2015–2019 vs 2020–2024 yielded consistent results (sHR 1.82 vs 1.89 for advanced stage, interaction *p* = 0.71), suggesting minimal temporal confounding; and although time-varying effects diagnostics were performed, formal Schoenfeld-type residual testing of the proportional subdistribution hazards assumption was performed (Supplementary Figure S3); no significant time-varying effects were detected for the primary covariates (global test *p* = 0.23), supporting the validity of the proportional hazards assumption over the 12-month analysis window. Furthermore, we could not disentangle the relative contributions of chemotherapy-related cognitive impairment (CRCI) and surgery-related perioperative cognitive decline, as adjuvant chemotherapy exposure during the follow-up period was not systematically captured in the current dataset. Given that CRCI shares pathophysiological mechanisms with perioperative neurocognitive disorders—including oxidative stress, neuroinflammation, and blood–brain barrier disruption [4,5]—the observed CCD events may represent a composite of both perioperative and chemotherapy-mediated cognitive injury. Prospective studies incorporating serial neuropsychological assessments before and after each treatment modality are needed to parse these overlapping contributions [29].

Three additional limitations merit explicit acknowledgment in light of peer review. First, the visit-frequency adjustment reduces but does not eliminate surveillance bias; unmeasured aspects of clinical attentiveness—oncologist vigilance, symptom-prompted referrals, family-initiated concerns—are not captured by raw outpatient visit counts, and part of the observed stage–CCD association may therefore reflect differential clinical recognition rather than a purely biological difference in underlying risk. Second, although we present cancer-type-stratified incidence and Fine–Gray estimates in Supplementary Table S12, our pooled primary analysis treats endometrial, ovarian, cervical, and other gynecologic cancers as a single population in order to preserve adequate events-per-variable; these cancers are biologically, therapeutically, and prognostically heterogeneous, and type-stratified prospective studies are needed to disentangle type-specific effect magnitudes. Third, factors such as postoperative delirium, intraoperative hypotension, and transfusion—reported in Table 4—are presented as perioperative factors associated with CCD; they may, however, function as markers of operative severity or postoperative clinical instability rather than as independent causal drivers, and any interpretation of “modifiability” is hypothesis-generating and requires prospective trial validation rather than direct clinical translation from the present observational data. The hypothesized causal structure supporting this interpretation is summarized in Supplementary Figure S4.

## 5. Conclusions

A positive association between advanced cancer stage (FIGO III–IV) and clinically significant cognitive decline was observed in gynecologic cancer patients undergoing surgery, independent of age and comorbidity burden. Competing-risk modeling is essential in this high-mortality population to avoid overestimation of CCD incidence. Older patients with advanced disease—particularly those aged ≥70 years—represent a high-risk phenotype that may benefit from targeted screening and preventive strategies, although these findings require validation in prospective multicenter cohorts with formal baseline cognitive assessment (e.g., Montreal Cognitive Assessment). Future prospective multicenter trials are needed to (i) validate the EMR-based CCD definition against formal neuropsychological testing, and (ii) evaluate ERAS-compatible delirium-prevention bundles in the ≥70 years + FIGO III–IV subgroup, where the present data suggest the highest potential clinical benefit. Recommendations regarding specific neuroprotective anesthetic strategies are beyond the scope of what the present retrospective design can support.

## Figures and Tables

**Table 1:** Baseline characteristics overall and by CCD status.

Variable	Overall (n = 1023)	CCD Yes (n = 98)	CCD No (n = 925)	SMD
Age (years), mean ± SD	62.4 ± 11.8	68.7 ± 9.9	61.6 ± 11.9	0.62
≥70 years, n (%)	312 (30.5)	48 (49.0)	264 (28.5)	0.43
CCI, median (IQR)	3 (2–5)	4 (3–6)	3 (2–4)	0.48
Advanced stage (III–IV), n (%)	423 (41.3)	58 (59.2)	365 (39.5)	0.42
Open surgery, n (%)	533 (52.1)	63 (64.3)	470 (50.8)	0.28
Postoperative delirium, n (%)	101 (9.9)	24 (24.5)	77 (8.3)	0.45

Data are presented as mean ± SD, median (IQR), or n (%). SMD > 0.1 indicates meaningful imbalance. **Abbreviations:** CCD: clinically significant cognitive decline; CCI: Charlson Comorbidity Index; IQR: interquartile range; SD: standard deviation; SMD: standardized mean difference.

**Table 2:** Incidence and competing events.

Outcome	n (%)	Median Time (IQR, Months)
CCD	98 (9.6)	4.8 (2.3–8.1)
All-cause death (competing)	140 (13.7)	5.2 (2.1–9.4)
Major neurologic event (stroke)	14	3.2 (1.8–5.9)
Hospice/terminal progression	23	6.1 (3.4–9.7)
12-month CIF of CCD (Fine–Gray)	—	11.2% (9.1–13.5)

Cumulative incidence estimated using Fine–Gray competing-risk models. For CIF, n(%) is not applicable; values are reported as % (95% CI). Abbreviation: CCD: clinically significant cognitive decline; CIF: cumulative incidence function; IQR: interquartile range.

**Table 3:** Multivariable Fine–Gray subdistribution hazard models for CCD.

Variable	Full Model sHR (95% CI)	*p*-Value
Advanced stage (III–IV)	1.85 (1.27–2.69)	0.001
Age (per 5-year increase)	1.29 (1.17–1.43)	<0.001
CCI (per 1-point)	1.14 (1.06–1.23)	<0.001
Age × stage interaction	—	<0.001

Adjusted for age (restricted cubic splines), CCI, cancer stage, and treatment intensity. Treatment intensity was defined as low (early-stage disease with minimally invasive surgery and no neoadjuvant chemotherapy), intermediate (any single intensity marker: advanced stage, neoadjuvant chemotherapy, or open surgery), and high (advanced stage plus at least one additional intensity marker). The age × stage interaction was tested by Wald χ^2^; a single sHR is not presented because the interaction involves a nonlinear (spline-modeled) age effect. Abbreviation: CCI: Charlson Comorbidity Index; CI: confidence interval; sHR: subdistribution hazard ratio.

**Table 4:** Perioperative factors associated with CCD.

Factor	sHR (95% CI)	*p*-Value
Open vs minimally invasive	1.54 (1.08–2.19)	0.017
Intraoperative hypotension	1.41 (1.02–1.95)	0.039
Transfusion	1.68 (1.12–2.52)	0.012
Postoperative delirium	2.76 (1.81–4.21)	<0.001

Estimates derived from multivariable Fine–Gray competing-risk models with prespecified covariate adjustment. Abbreviation: CI: confidence interval; sHR: subdistribution hazard ratio. These estimates should be interpreted as associations; hypotension, transfusion, and postoperative delirium may also function as markers of operative complexity or postoperative severity rather than direct causal targets.

## Data Availability

The data supporting the findings of this study are available from the corresponding authors upon reasonable request.

## Supplementary Materials

The following supporting information can be downloaded at: https://www.mdpi.com/article/10.3390/medicina62050988/s1, Table S1. Baseline Characteristics by CCD Status (n = 1023). Table S2. Complete-Case Analysis vs. Multiple Imputation Comparison. Table S3. Competing Events—Detailed Breakdown. Table S4. Visit-Frequency Adjusted Fine–Gray Model and Stage-Stratified Follow-up Density. Table S5. Univariable Fine–Gray Subdistribution Hazard Models for CCD. Table S6. Multivariable Fine–Gray Subdistribution Hazard Model for CCD (Primary Analysis). Table S7. Absolute 12-Month Risk of CCD by Cancer Stage (Cumulative Incidence Function). Table S8. Perioperative Potentially Modifiable Factors—IPTW-Adjusted Fine–Gray Models. Table S9. Cause-Specific Hazard Models for CCD (Secondary Analysis). Table S10. Sensitivity Analyses for Advanced Stage Association with CCD. Table S11. Sensitivity Analysis—POD as Mediator versus Confounder in the Core Fine–Gray Model. Table S12. Cancer-Type-Stratified 12-Month CCD Incidence and Advanced-Stage Fine–Gray Models. Table S13. Characteristics of the Multi-Criterion CCD Subgroup. Figure S1. Interaction Plot: Age × Stage Effect on Predicted 12-Month CCD Risk. Figure S2. Calibration Plot with Bootstrap Optimism Correction (Internal Validation). Figure S3. Schoenfeld-type Residual Diagnostics for the Proportional Subdistribution Hazards Assumption. Figure S4. Directed Acyclic Graph (DAG) of the Hypothesised Causal Structure. Supplementary Material S1. Standardized Data Dictionary and Prespecified Variable Definitions. Supplementary Material S2: Completed STROBE Checklist. Appendix S1: R Code for Supplementary Analyses.

## Abbreviations

The following abbreviations are used in this manuscript:

BMI	Body mass index
CCD	Clinically significant cognitive decline
CCI	Charlson Comorbidity Index
CI	Confidence interval
CRCI	Chemotherapy-related cognitive impairment
EMR	Electronic medical record
ERAS	Enhanced Recovery After Surgery
FIGO	International Federation of Gynecology and Obstetrics
ICD-10	International Classification of Diseases, 10th Revision
IOH	Intraoperative hypotension
IPTW	Inverse probability of treatment weighting
IQR	Interquartile range
IRB	Institutional Review Board
MAP	Mean arterial pressure
NPV	Negative predictive value
POD	Postoperative delirium
POCD	Postoperative cognitive dysfunction
PPV	Positive predictive value
RD	Risk difference
sHR	Subdistribution hazard ratio
SMD	Standardized mean difference
STROBE	Strengthening the Reporting of Observational Studies in Epidemiology
