# Supplementary material for: Association of Cancer Stage and Comorbidity Burden with 12-Month Clinically Significant Cognitive Decline After Gynecologic Cancer Surgery: A Competing-Risk Retrospective Cohort Study
Source: Medicina (Kaunas). 2026 May 19;62(5):988. doi: 10.3390/medicina62050988 (PMC13208514; doi:10.3390/medicina62050988)
Supplement: Supplementary file 1 [file medicina-62-00988-s001.zip › Supplementary Material S1 Standardized Data Dictionary.pdf]

Supplementary Material S1. Standardized Data Dictionary and Prespecified Variable Definitions

Study title: Association of Cancer Stage and Comorbidity Burden With 12-Month Clinically Significant Cognitive Decline After Gynecologic Cancer Surgery: A Competing-Risk Retrospective Cohort Study

- All variables listed below were prespecified before final model fitting and were used to standardize extraction from the EMR, AIMS, laboratory information system, and institutional oncology registry.
- The index date was defined as the date of the index gynecologic cancer surgery, with start of anesthesia used as time zero for time-to-event analyses.
- The core Fine-Gray model included age, CCI, cancer stage, and treatment intensity. Perioperative factors were evaluated as associative signals or sensitivity exposures, not as validated causal treatment targets.
- Where exact dates were unavailable for a qualifying event, the first date of clinical documentation in the EMR was used.

Data dictionary

Variable / domain	Operational definition	Coding / analysis handling	Source / timing
**Analytic cohort**	Adult women undergoing index surgery for histologically confirmed gynecologic cancer at Wonkwang University Hospital during 1 January 2015 to 31 December 2024, after applying all prespecified inclusion and exclusion criteria.	Binary eligibility indicator; included/excluded with reason.	EMR, AIMS, oncology registry; assessed before database lock.
**Index date / time zero**	Date of index gynecologic cancer surgery; start of anesthesia was used as the time origin for time-to-event analyses.	Calendar date; day 0 for follow-up.	AIMS and operative record.
**Follow-up time**	Time from index date to the earliest of CCD event, competing event, loss to follow-up, or administrative censoring.	Days, converted to months for descriptive reporting; primary horizon 365 days; extended sensitivity horizon 24 months.	EMR encounter history, mortality data, oncology registry.
**Age**	Age at index surgery.	Continuous in years; modelled with restricted cubic splines in primary models; also categorized as <70 versus >=70 years for prespecified subgroup and interaction presentation.	Demographic record at index admission.
**Sex**	Biologic sex recorded in the EMR.	Female only by cohort definition.	Demographic record.
**Body mass index**	Weight divided by height squared using the closest preoperative measurement.	kg/m2; descriptive covariate.	Preoperative nursing/anesthesia record.
**Charlson Comorbidity Index (CCI)**	Comorbidity burden calculated from diagnosis codes and problem lists in the 12-month preoperative lookback window.	Continuous integer score; also summarized categorically when needed (e.g., CCI >=4).	ICD-10 codes, EMR problem list, preoperative assessment.
**Pre-existing cognitive impairment**	Evidence of dementia, neurodegenerative disease, or chronic cognition-targeted therapy before surgery.	Exclusion variable: documented dementia/neurodegenerative disease or chronic cholinesterase inhibitor/memantine use during the 12-month lookback window.	ICD-10 codes, neurology/psychiatry problem list, medication records.
**Cancer type**	Primary gynecologic cancer site/histology category.	Categorical: endometrial, ovarian, cervical, or other (including vulvar and primary peritoneal carcinoma).	Pathology report and oncology registry.
**Cancer stage**	Cancer stage at the time of index surgery according to International Federation of Gynecology and Obstetrics (FIGO) staging.	Primary coding: FIGO I-II versus FIGO III-IV; advanced stage defined as III-IV.	Oncology registry, operative/pathology reports.
**Neoadjuvant chemotherapy**	Receipt of systemic chemotherapy before index surgery.	Binary: yes/no.	Oncology registry, chemotherapy orders, EMR treatment history before index date.
**Adjuvant chemotherapy during follow-up**	Systemic chemotherapy delivered after surgery during the 12-month follow-up period.	Not entered as a time-varying covariate because historical oncology pharmacy records were not linkable at patient level across the full study window; considered an unmeasured mediator/overlapping pathway in the DAG and limitations.	Oncology pharmacy system not linkable to analytic file for the full 2015-2024 period.
**Surgical approach**	Primary operative approach for the index cancer surgery.	Binary: open versus minimally invasive surgery (laparoscopic or robotic).	Operative note and procedure codes.
**Treatment intensity**	Composite ordinal marker	Low: early-stage disease with	Derived from stage, neoadjuvant

Variable / domain	Operational definition	Coding / analysis handling	Source / timing
	summarizing stage, neoadjuvant therapy, and operative approach.	minimally invasive surgery and no neoadjuvant chemotherapy; intermediate: any single intensity marker (advanced stage, neoadjuvant chemotherapy, or open surgery); high: advanced stage combined with at least one additional intensity marker.	chemotherapy, and surgical approach.
**Operative time**	Elapsed time from incision/procedure start to closure/procedure end, or anesthesia-record operative duration when operative timestamps were unavailable.	Minutes; descriptive and sensitivity covariate.	AIMS and operative record.
**Estimated blood loss**	Estimated intraoperative blood loss documented by the anesthesia or operative team.	mL; summarized descriptively.	AIMS and operative record.
**Intraoperative hypotension burden**	Burden of intraoperative mean arterial pressure below 65 mmHg.	Primary extraction: time-weighted MAP <65 mmHg exposure; tabulated as presence/absence of hypotension burden and used in sensitivity models as recorded in the analysis dataset.	AIMS minute-by-minute or interval blood-pressure records.
**Transfusion**	Any intraoperative or immediate perioperative blood-product transfusion related to the index surgery.	Binary: yes/no; product-specific details extracted when available.	AIMS, blood bank record, operative/anesthesia notes.
**Opioid dose**	Total opioid exposure recorded during the index perioperative episode.	Dose harmonized to opioid-equivalent units according to institutional conversion rules; evaluated as a perioperative exposure when available and not included in the core competing-risk model.	AIMS medication administration record and inpatient medication record.
**Postoperative delirium (POD)**	Clinically documented delirium within 7 postoperative days.	Binary: yes/no. Treated as a perioperative factor and, in causal interpretation, as a mediator on the pathway from perioperative severity/intraoperative exposures to CCD rather than as a core confounder.	CAM/CAM-ICU documentation when available, physician/nursing notes, diagnosis codes, and medication/consultation evidence.
**Clinically significant cognitive decline (CCD)**	Primary outcome defined as the earliest EMR-documented event indicating clinically recognized cognitive decline occurring >=30 days after surgery: new clinician diagnosis of cognitive impairment, neurology/psychiatry consultation for cognitive concerns, or initiation of cognition-targeted pharmacotherapy.	Time-to-event outcome; event date was the first qualifying documentation date. Events before postoperative day 30 were not counted as CCD to reduce conflation with acute delirium.	EMR diagnoses/free text, consultation records, medication records.
**CCD component criteria**	Individual components of the composite CCD outcome.	Three non-mutually exclusive binary indicators: clinician diagnosis, neurology/psychiatry consultation, and cognition-targeted pharmacotherapy. Patients meeting >=2 criteria were characterized as a multi-criterion CCD subgroup.	EMR review and structured extraction.
**Competing event**	Event precluding subsequent reliable observation of CCD before the end of follow-up.	All-cause death, major neurologic event (stroke) precluding assessment, hospice enrollment, or terminal cancer progression; mutually exclusive hierarchy applied according to earliest event date.	Mortality record, EMR, oncology registry, discharge/hospice documentation.
**Censoring**	No CCD or competing event before the end of observed follow-up.	Censored at last eligible follow-up encounter or administrative end of the prespecified follow-up period.	EMR encounter history and oncology registry.
**Outpatient visit frequency**	Intensity of postoperative clinical surveillance during follow-up.	Total 0-12 month outpatient encounters and contacts per patient-month; added in a sensitivity model to address differential surveillance by stage.	EMR encounter logs.
**Age x stage interaction**	Prespecified effect modification between age and advanced cancer stage.	Wald chi-square test in Fine-Gray model; presented using spline-based prediction and age-stratified cumulative incidence curves.	Derived model term.
**Age x CCI interaction**	Prespecified effect modification between age and comorbidity burden.	Wald chi-square test in Fine-Gray model.	Derived model term.

Variable / domain	Operational definition	Coding / analysis handling	Source / timing
**Missing data**	Missingness in key variables before imputation.	Multiple imputation by chained equations with 10 imputed datasets and Rubin's rules; complete-case analysis performed as a sensitivity analysis.	Analysis dataset.
**Validation sample**	Stratified random chart-review sample used to evaluate the EMR-based CCD definition.	80 flagged-positive and 80 unflagged controls; blinded duplicate review against structured reference standard; PPV, sensitivity, specificity, NPV, and kappa reported.	Random sample from full analytic cohort and blinded chart review.

Abbreviations: AIMS, anesthesia information management system; CAM, Confusion Assessment Method; CAM-ICU, Confusion Assessment Method for the Intensive Care Unit; CCD, clinically significant cognitive decline; CCI, Charlson Comorbidity Index; EMR, electronic medical record; FIGO, International Federation of Gynecology and Obstetrics; MAP, mean arterial pressure; NPV, negative predictive value; POD, postoperative delirium; PPV, positive predictive value.

End of Supplementary Material S1.
