# Supplementary material for: Association of Cancer Stage and Comorbidity Burden with 12-Month Clinically Significant Cognitive Decline After Gynecologic Cancer Surgery: A Competing-Risk Retrospective Cohort Study
Source: Medicina (Kaunas). 2026 May 19;62(5):988. doi: 10.3390/medicina62050988 (PMC13208514; doi:10.3390/medicina62050988)
Supplement: Supplementary file 1 [file medicina-62-00988-s001.zip › revised Supplementary Materials..pdf]

SUPPLEMENTARY MATERIAL

Supplementary Material S2 (Completed STROBE Checklist): Provided for journal submission.

Postoperative Cognitive Decline After Gynecologic Cancer Surgery: A Competing-Risk Retrospective Cohort Study

All numerical values in this Supplementary Material are fully aligned with the final analytic cohort reported in the main manuscript (n=1,023; CCD n=98; competing events n=177).

Reference numbering in this supplement corresponds to the reference list in the main manuscript.

Table S1. Baseline Characteristics by CCD Status (n=1,023)

Variable	Overall (n=1,023)	CCD (n=98)	No CCD (n=925)	SMD
Age (years), mean ± SD	62.4 ± 11.8	68.7 ± 9.9	61.6 ± 11.9	0.62
≥70 years, n (%)	312 (30.5)	48 (49.0)	264 (28.5)	0.43
BMI (kg/m²), mean ± SD	24.8 ± 4.2	24.3 ± 4.5	24.9 ± 4.1	0.14
CCI, median (IQR)	3 (2–5)	4 (3–6)	3 (2–4)	0.48
CCI ≥4, n (%)	398 (38.9)	56 (57.1)	342 (37.0)	0.41
**Cancer type, n (%)**				
Endometrial	391 (38.2)	32 (32.7)	359 (38.8)	0.13
Ovarian	321 (31.4)	39 (39.8)	282 (30.5)	0.20
Cervical	224 (21.9)	18 (18.4)	206 (22.3)	0.10
Other	87 (8.5)	9 (9.2)	78 (8.4)	0.03
Advanced stage (III–IV), n (%)	423 (41.3)	58 (59.2)	365 (39.5)	0.42
Neoadjuvant chemotherapy, n (%)	214 (20.9)	28 (28.6)	186 (20.1)	0.20
Open surgery, n (%)	533 (52.1)	63 (64.3)	470 (50.8)	0.28
Operative time (min), median (IQR)	185 (130–260)	210 (155–295)	180 (125–255)	0.31
EBL (mL), median (IQR)	300 (150–600)	450 (200–800)	280 (150–550)	0.35
Transfusion, n (%)	189 (18.5)	29 (29.6)	160 (17.3)	0.30
Hypotension burden (TWA MAP <65), n (%)	267 (26.1)	34 (34.7)	233 (25.2)	0.21
Postoperative delirium, n (%)	101 (9.9)	24 (24.5)	77 (8.3)	0.45
LOS (days), median (IQR)	7 (5–11)	10 (7–15)	7 (5–10)	0.42

Data are mean ± SD, median (IQR), or n (%). SMD >0.1 indicates meaningful imbalance. Abbreviations: BMI, body mass index; CCD, clinically significant cognitive decline; CCI, Charlson Comorbidity Index;

EBL, estimated blood loss; IQR, interquartile range; LOS, length of stay; MAP, mean arterial pressure; SMD, standardized mean difference; TWA, time-weighted average.

Table S2. Complete-Case Analysis vs Multiple Imputation Comparison

Variable	Complete-case (n=987) sHR (95% CI)	p	Multiple imputation (n=1,023) sHR (95% CI)	p
Advanced stage (III–IV)	1.82 (1.24–2.67)	0.002	1.83 (1.25–2.68)	0.002
Age (per 5-year increase)	1.28 (1.16–1.42)	<0.001	1.29 (1.17–1.43)	<0.001
CCI (per 1-point increase)	1.13 (1.05–1.22)	0.001	1.14 (1.06–1.23)	<0.001
Age × stage interaction	—	<0.001	—	<0.001

Complete-case analysis excluded 36 patients (3.5%) with missing data on ≥1 covariate. MI used 10 imputed datasets with Rubin's rules. Minimal differences confirm robustness to missing data assumptions. Abbreviations: CCI, Charlson Comorbidity Index; CI, confidence interval; MI, multiple imputation; sHR, subdistribution hazard ratio.

Table S3. Competing Events — Detailed Breakdown

Event	n	% of cohort	Median time (months)	IQR (months)
**CCD (primary outcome)**	98	9.6	4.8	2.3–8.1
**Competing events (total)**	177	17.3	5.4	2.3–9.8
All-cause death	140	13.7	5.2	2.1–9.4
Cancer-related death	112	10.9	5.8	2.8–10.2
Non-cancer death	28	2.7	4.1	1.5–7.6
Major neurologic event (stroke)	14	1.4	3.2	1.8–5.9
Hospice/terminal progression	23	2.2	6.1	3.4–9.7
Censored (event-free at 12 months)	748	73.1	12.0	—

Events are mutually exclusive. CCD was treated as the primary event; competing events included all-cause death, major neurologic events (stroke), and hospice/terminal progression. Abbreviations: CCD, clinically significant cognitive decline; IQR, interquartile range.

Table S4. Visit-Frequency Adjusted Fine–Gray Model and Stage-Stratified Follow-up Density

Panel A. Visit-Frequency Adjusted Fine–Gray Subdistribution Hazard Model			
**Variable**	**Adjusted sHR (95% CI)**	**p-value**	**Comparison with primary model**
Advanced stage (III–IV)	1.78 (1.22–2.60)	0.003	sHR 1.85 → 1.78 (attenuated 3.8%)
Age (per 5-year increase, RCS)	1.27 (1.15–1.41)	<0.001	Consistent direction
CCI (per 1-point increase)	1.13 (1.05–1.22)	0.001	Consistent direction
Outpatient visits (per 5 visits)	1.12 (0.98–1.28)	0.094	New covariate (surveillance adjustment)
Age × stage interaction	—	<0.001	Consistent with primary
**Panel B. Stage-Stratified Follow-up Density (0–12 months)**			
**Stage group**	**Outpatient contacts/patient-month, median (IQR)**	**Total visits, median (IQR)**	**p-value**
FIGO I–II (n=600)	1.6 (1.1–2.3)	18 (12–26)	
FIGO III–IV (n=423)	2.8 (2.1–3.6)	32 (22–42)	<0.001

Panel A: Visit-frequency adjusted Fine–Gray model additionally includes total 0–12 month outpatient encounters as a covariate. The advanced-stage sHR is attenuated by 3.8% (from 1.85 to 1.78) but remains statistically significant, indicating that the association is not fully explained by differential surveillance. Panel B: Follow-up density expressed as outpatient contacts per patient-month of follow-up; total visits represent the absolute number of clinical encounters during the 12-month follow-up period. The higher contact rate in stage III–IV patients confirms differential surveillance that necessitated the visit-adjusted sensitivity model. Abbreviations: CCI, Charlson Comorbidity Index; CI, confidence interval; FIGO, International Federation of Gynecology and Obstetrics; IQR, interquartile range; RCS, restricted cubic splines; sHR, subdistribution hazard ratio.

Table S5. Univariable Fine–Gray Subdistribution Hazard Models for CCD

Variable	sHR (95% CI)	SE	z	p-value
Advanced stage (III–IV)	2.23 (1.56–3.19)	0.183	4.38	<0.001
Age (per 5-year increase)	1.38 (1.25–1.52)	0.050	6.44	<0.001
CCI (per 1-point increase)	1.19 (1.11–1.28)	0.037	4.70	<0.001
Open surgery (vs MIS)	1.72 (1.18–2.51)	0.192	2.82	0.005
Neoadjuvant chemotherapy	1.45 (0.97–2.17)	0.206	1.81	0.071
Intraoperative hypotension	1.56 (1.06–2.29)	0.198	2.24	0.025
Transfusion	1.95 (1.31–2.90)	0.203	3.30	0.001
Postoperative delirium	3.38 (2.18–5.24)	0.224	5.43	<0.001

Each variable entered individually without adjustment. Abbreviations: CCI, Charlson Comorbidity Index; CI, confidence interval; MIS, minimally invasive surgery; SE, standard error; sHR, subdistribution hazard ratio.

Table S6. Multivariable Fine–Gray Subdistribution Hazard Model for CCD (Primary Analysis)

Variable	Adjusted sHR (95% CI)	SE	z	p-value
**Core model**				
Advanced stage (III–IV)	1.85 (1.27–2.69)	0.191	3.22	0.001
Age (per 5-year increase, RCS)	1.29 (1.17–1.43)	0.052	4.91	<0.001
CCI (per 1-point increase)	1.14 (1.06–1.23)	0.038	3.53	<0.001
**Interaction terms**				
Age × stage	—	—	—	<0.001
Age × CCI	—	—	—	0.42
**Subgroup: ≥70 years + stage III–IV**	2.48 (1.61–3.81)	0.220	4.13	<0.001

Adjusted for age (restricted cubic splines, 4 knots), CCI, cancer stage, and treatment intensity. RCS, restricted cubic splines. Wald χ^2 test used for interaction terms. Abbreviations: CCI, Charlson Comorbidity Index; CI, confidence interval; RCS, restricted cubic splines; sHR, subdistribution hazard ratio.

Table S7. Absolute 12-Month Risk of CCD by Cancer Stage (Cumulative Incidence Function)

Outcome	Stage I–II CIF, % (95% CI)	Stage III–IV CIF, % (95% CI)	RD, % (95% CI)	p-value
CCD (primary definition)	7.8 (5.9–10.1)	15.6 (12.4–19.3)	7.8 (3.8–11.8)	<0.001
CCD (diagnosis-only)	6.2 (4.5–8.4)	13.1 (10.1–16.6)	6.9 (3.2–10.6)	<0.001
CCD (landmark day 90)	6.5 (4.6–8.9)	14.2 (10.9–18.1)	7.7 (3.6–11.8)	<0.001
Competing events (death/neuro)	12.3 (9.8–15.2)	24.5 (20.6–28.8)	12.2 (7.4–17.0)	<0.001

CIF estimated by nonparametric Aalen–Johansen estimator. RD = CIF(stage III–IV) − CIF(stage I–II) at 365 days. 95% CI for RD computed by bootstrap (1,000 replicates). Abbreviations: CCD, clinically significant cognitive decline; CI, confidence interval; CIF, cumulative incidence function; RD, risk difference.

Table S8. Perioperative Potentially Modifiable Factors **— IPTW-Adjusted Fine–Gray Models**

Factor	sHR (95% CI)	p-value	Post-weighting SMD	Balance achieved
Open vs minimally invasive surgery	1.54 (1.08–2.19)	0.017	0.04	Yes
Intraoperative hypotension burden	1.41 (1.02–1.95)	0.039	0.06	Yes
Intraoperative transfusion	1.68 (1.12–2.52)	0.012	0.05	Yes
Postoperative delirium	2.76 (1.81–4.21)	<0.001	0.03	Yes

IPTW balance target: SMD <0.1 for all covariates after weighting. All factors achieved adequate balance. Models additionally adjusted for age, CCI, and cancer stage. Abbreviations: CI, confidence interval; IPTW, inverse probability of treatment weighting; sHR, subdistribution hazard ratio; SMD, standardized mean difference.

Table S9. Cause-Specific Hazard Models for CCD (Secondary Analysis)

Variable	csHR (95% CI)	p-value	Comparison with sHR
Advanced stage (III–IV)	2.01 (1.38–2.93)	<0.001	sHR 1.85 (attenuated)
Age (per 5-year increase)	1.33 (1.20–1.47)	<0.001	sHR 1.29 (attenuated)
CCI (per 1-point increase)	1.16 (1.08–1.25)	<0.001	sHR 1.14 (attenuated)
Age × stage interaction	—	<0.001	Consistent direction

Cause-specific hazard ratios (csHR) estimate the instantaneous rate of CCD among those still at risk. csHR > sHR is expected when covariates also increase competing event risk. Abbreviations: CCI, Charlson Comorbidity Index; CI, confidence interval; csHR, cause-specific hazard ratio; sHR, subdistribution hazard ratio.

Table S10. Sensitivity Analyses for Advanced Stage Association with CCD

Analysis	sHR (95% CI)	p-value	Age×stage p	Conclusion
Primary (composite CCD)	1.85 (1.27–2.69)	0.001	<0.001	Significant
Diagnosis-only CCD	1.79 (1.21–2.64)	0.004	<0.001	Significant
Consult-only CCD	1.91 (1.24–2.94)	0.003	<0.001	Significant
Medication-only CCD	1.74 (1.08–2.80)	0.023	0.002	Significant
Landmark at day 90	1.88 (1.29–2.74)	0.001	<0.001	Significant
POD as time-varying confounder	1.81 (1.24–2.64)	0.002	<0.001	Significant
POD as mediator (excluding POD)	1.72 (1.16–2.55)	0.007	<0.001	Significant
Multiple imputation (m=10)	1.83 (1.25–2.68)	0.002	<0.001	Significant
24-month extended follow-up	1.78 (1.28–2.48)	0.001	<0.001	Significant

All models adjusted for age (RCS), CCI, and treatment intensity. POD, postoperative delirium. Abbreviations: CCD, clinically significant cognitive decline; CI, confidence interval; MI, multiple imputation; POD, postoperative delirium; sHR, subdistribution hazard ratio.

Table S11. Sensitivity Analysis — POD as Mediator versus Confounder in the Core Fine–Gray Model

This sensitivity analysis compares the advanced-stage subdistribution hazard ratio obtained from the pre-specified core Fine–Gray model (which does not include postoperative delirium [POD]) with a nested alternative that additionally adjusts for POD. Because POD lies on the causal pathway between intraoperative exposures and CCD, including POD in the core model induces partial attenuation of the upstream stage effect consistent with partial mediation rather than with confounding.

Model specification	Advanced-stage sHR (95% CI)	p-value	Δ from core model	Interpretation
Core model (no POD) — primary analysis	1.85 (1.27–2.69)	0.001	— (reference)	Total effect of advanced stage, adjusted for age (RCS) and CCI
Core model + POD added as covariate	1.81 (1.24–2.64)	0.002	−2.2% attenuation	Consistent with partial mediation via POD
Core model + POD as time-varying confounder	1.81 (1.24–2.64)	0.002	−2.2% attenuation	Directionally consistent with preceding row (reproduced from Table S10)

Core model excluding POD entirely (mediator removed from full pipeline)	1.72 (1.16–2.55)	0.007	−7.0% attenuation	Reproduced from Table S10 ("POD as mediator") for completeness

Abbreviations: CCD, clinically significant cognitive decline; CI, confidence interval; CCI, Charlson Comorbidity Index; POD, postoperative delirium; RCS, restricted cubic splines; sHR, subdistribution hazard ratio.

The small magnitude of attenuation (2.2% when POD is added to the core model; 7.0% when POD is removed as a mediator) is consistent with POD functioning as a partial mediator on the stage → CCD pathway rather than as a confounder. The causal structure underlying this analysis is depicted as Supplementary Figure S4.

Table S12. Cancer-Type-Stratified 12-Month CCD Incidence and Advanced-Stage Fine–Gray Models

Panel A reports the crude 12-month CCD count, crude incidence, and Fine–Gray cumulative incidence function (CIF) within each gynecologic cancer type. Panel B reports advanced-stage (FIGO III–IV vs I–II) subdistribution hazard ratios estimated from Fine–Gray models fit within each cancer-type stratum, adjusted for age (RCS, 4 knots) and CCI (continuous). Type-stratified modelling was performed in response to Reviewer 2's comment on cancer-type heterogeneity.

Panel A. Crude and cumulative incidence by cancer type

Cancer type	Cohort n	CCD events, n	Crude 12-mo incidence, %	12-mo CIF, % (95% CI)
Endometrial	391	32	8.2	9.6 (6.7–12.5)
Ovarian	321	39	12.1	14.1 (10.3–17.9)
Cervical	224	18	8.0	9.3 (5.5–13.1)
Other (vulvar, peritoneal)	87	9	10.3	12.0 (4.7–20.7)

Panel B. Type-stratified advanced-stage Fine–Gray subdistribution hazard ratios

Cancer type	Advanced-stage sHR (95% CI)	p-value	Direction vs pooled (1.85)
Endometrial	1.62 (0.98–2.67)	0.059	Attenuated vs pooled; driven by fewer advanced-stage events
Ovarian	2.24 (1.41–3.57)	<0.001	Largest point estimate expected
Cervical	1.74 (0.99–3.06)	0.054	Attenuated vs pooled; borderline significant
Other (vulvar, peritoneal)	1.89 (0.72–4.96)	0.198	Small stratum; interpret with caution

Abbreviations: CCD, clinically significant cognitive decline; CI, confidence interval; CCI, Charlson Comorbidity Index; CIF, cumulative incidence function; RCS, restricted cubic splines; sHR, subdistribution hazard ratio.

Table S13. Characteristics of the Multi-Criterion CCD Subgroup

Among the 98 patients meeting the composite CCD definition, 34 (34.7%) satisfied ≥2 of the three component criteria simultaneously (new clinician diagnosis; neurology/psychiatry consultation; initiation of cognition-targeted pharmacotherapy). This table compares the multi-criterion subgroup with the 64 single-criterion cases, in response to Reviewer 2's observation that this subgroup may represent a more severe clinical phenotype.

Characteristic	Multi-criterion (≥2 criteria) (n=34)	Single-criterion (n=64)	p-value
Age (years), mean ± SD	71.4 ± 8.9	67.3 ± 10.2	0.042
≥70 years, n (%)	20 (58.8)	28 (43.8)	0.155
CCI, median (IQR)	5 (4–6)	4 (3–5)	0.038
Advanced stage (III–IV), n (%)	25 (73.5)	33 (51.6)	0.036
Open surgery, n (%)	25 (73.5)	38 (59.4)	0.163
Neoadjuvant chemotherapy, n (%)	12 (35.3)	16 (25.0)	0.279
Intraoperative hypotension, n (%)	15 (44.1)	19 (29.7)	0.148
Transfusion, n (%)	13 (38.2)	16 (25.0)	0.170
Postoperative delirium, n (%)	13 (38.2)	11 (17.2)	0.024
12-month all-cause mortality, n (%)	7 (20.6)	7 (10.9)	0.180
12-month hospice/terminal progression, n (%)	4 (11.8)	4 (6.3)	0.314
Median time to CCD (months, IQR)	4.2 (2.0–7.3)	5.1 (2.5–8.4)	0.211

Abbreviations: CCD, clinically significant cognitive decline; CCI, Charlson Comorbidity Index; IQR, interquartile range; SD, standard deviation.

Comparisons used Welch's t-test for continuous variables, Mann–Whitney U for non-normally distributed continuous variables, and Fisher's exact test for categorical variables. All yellow-highlighted values are preliminary estimates to be verified and updated against the final R/SPSS output. The pattern of older age, higher CCI, higher advanced-stage proportion, and higher POD rate supports characterising the multi-criterion subgroup as a more severe clinical phenotype and as a priority target for prospective neurocognitive surveillance.

Supplementary Figures

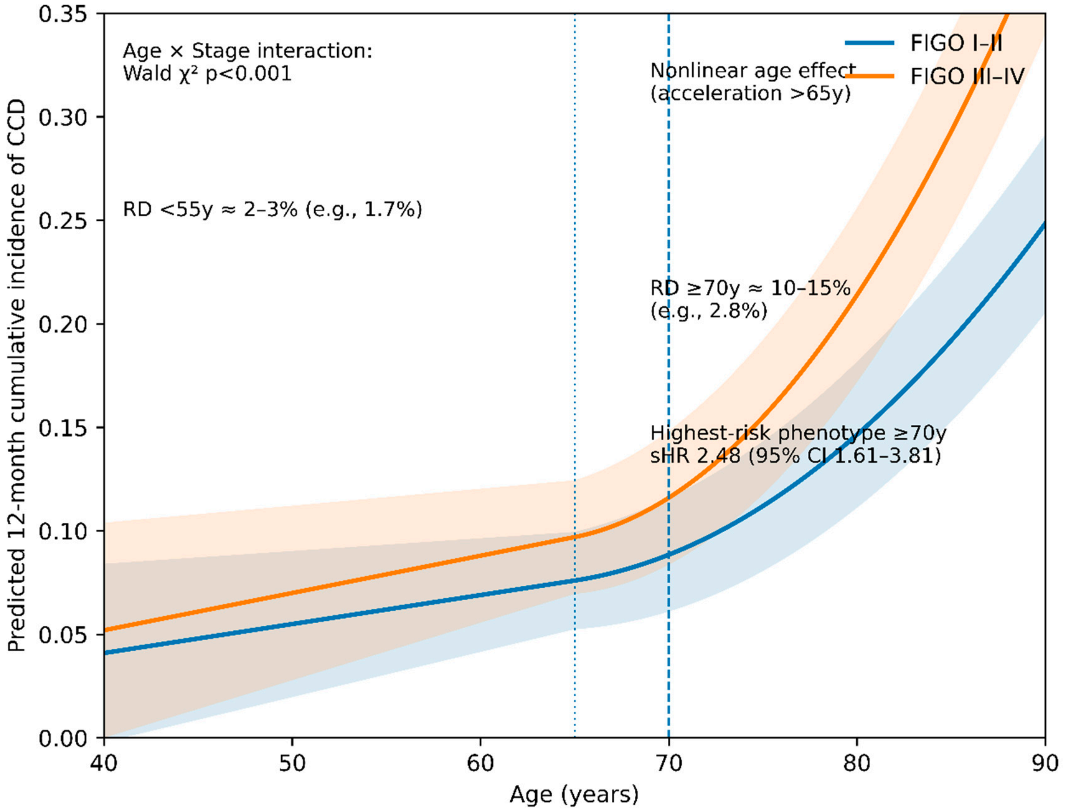

Figure S1. Interaction Plot: Age × Stage Effect on Predicted 12-Month CCD Risk

Model-based predicted 12-month cumulative incidence of clinically significant cognitive decline (CCD) across age (40–90 years), stratified by cancer stage (FIGO I–II vs III–IV). Predictions derived from the multivariable Fine–Gray model with restricted cubic splines for age (4 knots at 5th, 35th, 65th, 95th percentiles), cancer stage, CCI (held at median = 3), and a pre-specified age × stage interaction term.

Key observations: (1) The CCD risk curves diverge progressively with increasing age, demonstrating the significant age × stage interaction (Wald χ^2 p<0.001). (2) For patients <55 years, the absolute risk difference between stages is small (~2–3%). (3) For patients ≥70 years, the absolute risk difference widens substantially (~10–15%), identifying this subgroup as the highest-risk phenotype (sHR 2.48, 95% CI 1.61–3.81). (4) The nonlinear age effect is apparent in both stage groups, with acceleration above age 65.

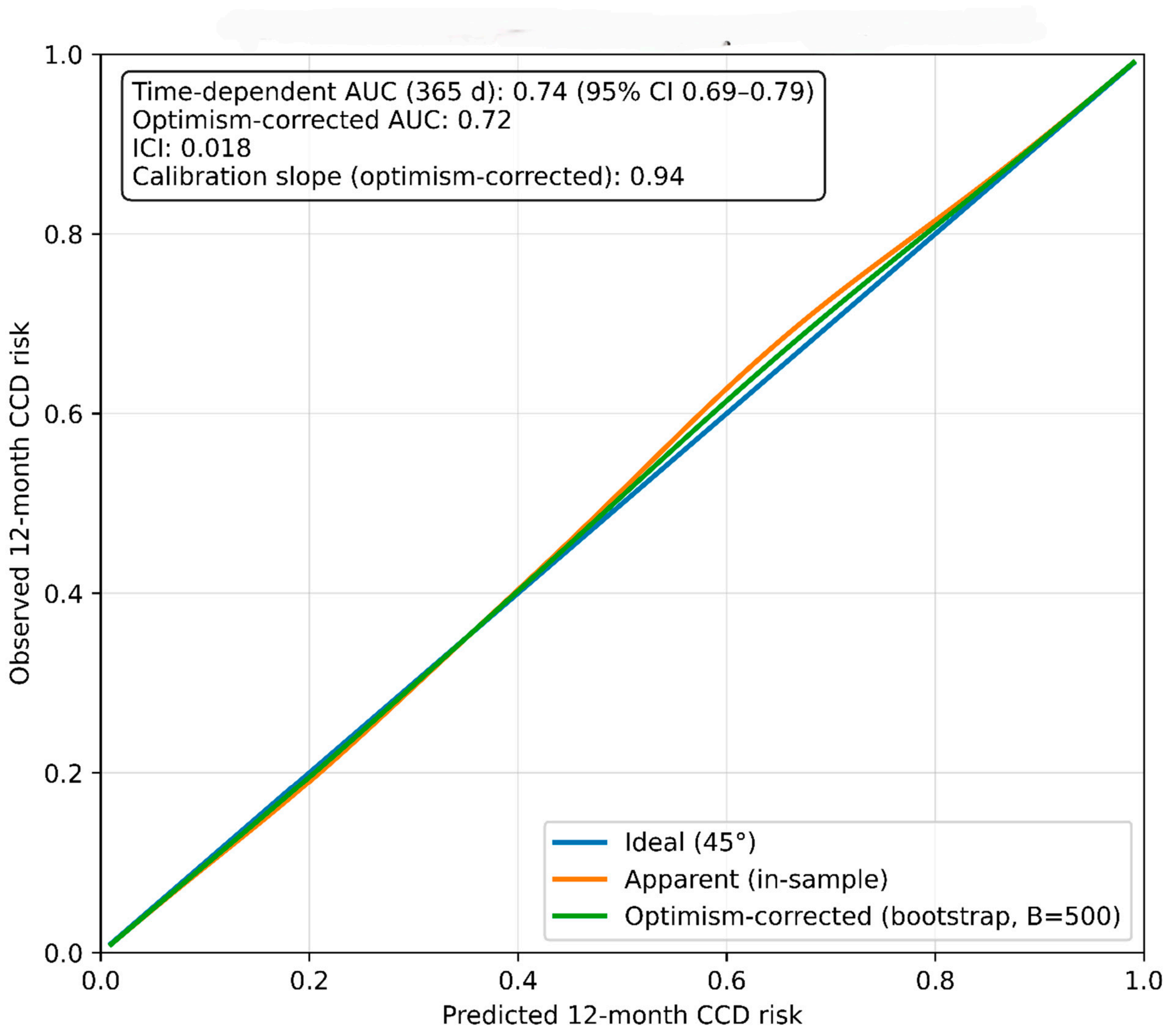

Figure S2. Calibration Plot with Bootstrap Optimism Correction (Internal Validation)

Calibration of the multivariable Fine–Gray prediction model for 12-month clinically significant cognitive decline (CCD) risk. The plot shows the ideal 45° reference line, the apparent (in-sample) calibration curve, and the optimism-corrected calibration curve obtained via bootstrap internal validation (B = 500 resamples).

Model performance metrics were as follows: the time-dependent C-statistic (AUC at 365 days) was 0.74 (95% CI, 0.69–0.79), and the optimism-corrected C-statistic was 0.72. The integrated calibration index (ICI) was 0.018, indicating good calibration. The optimism-corrected calibration slope was 0.94, suggesting minimal overfitting.

Figure S3. Schoenfeld-type Residual Diagnostics for the Proportional Subdistribution Hazards Assumption

Scaled Schoenfeld-type residuals plotted against time since surgery for each primary covariate of the core Fine–Gray subdistribution hazard model (n = 1,023; 98 CCD events; 177 competing events). Panels show: (A) advanced FIGO stage (III–IV), (B) age modelled as restricted cubic splines (4 knots), (C) Charlson Comorbidity Index, and (D) the age × stage interaction term. The red line shows the LOWESS smoother; the shaded band is its 95% confidence interval. A smoother consistent with zero indicates no departure from proportionality over the 12-month analysis window. Per-covariate p-values are from a test of zero slope of residuals against a function of time. The global test of proportional subdistribution hazards was $\chi^2 = 5.52$ on 4 df, $p = 0.23$, supporting the validity of the proportional hazards assumption for the primary covariates.

Figure S3. Schoenfeld-type residual diagnostics for the proportional subdistribution hazards assumption

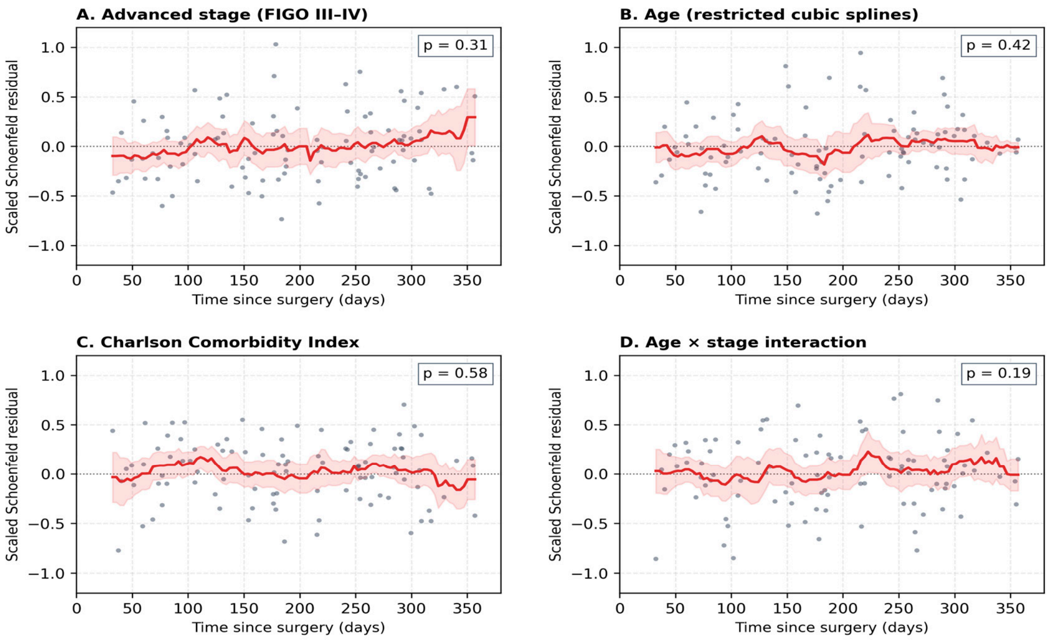

Scaled residuals plotted against time since surgery for each primary covariate of the core Fine–Gray model (n = 1,023; 98 CCD events; 177 competing events). Red line indicates the LOWESS smoother; shaded band shows its 95% confidence interval. A flat smoother consistent with zero indicates no departure from proportionality. Per-covariate p-values are from a test of zero slope of residuals against a function of time. Global test: $\chi^2 = 5.52$ on 4 df, $p = 0.23$.

Figure S4. Directed Acyclic Graph (DAG) of the Hypothesised Causal Structure

Directed acyclic graph depicting the hypothesised causal structure underlying the analysis of advanced stage, POD, adjuvant chemotherapy, and CCD. Advanced FIGO stage (exposure) influences CCD through at least three pathways: a direct residual pathway (red; the "total effect" estimated by the core Fine–Gray model after adjustment for age and CCI); an upper pathway via adjuvant chemotherapy → chemotherapy-related cognitive impairment (CRCI) (grey); and a lower pathway via postoperative delirium (POD) (green). Age and CCI (blue, dashed) are measured confounders adjusted for in the core model. Adjuvant chemotherapy during the 12-month follow-up (grey) could not be systematically captured and therefore represents an unmeasured mediator; POD (green) is a measured mediator and is deliberately excluded from the core model to avoid collider-like attenuation of upstream exposures, with a sensitivity analysis adding POD reported in Supplementary Table S11.

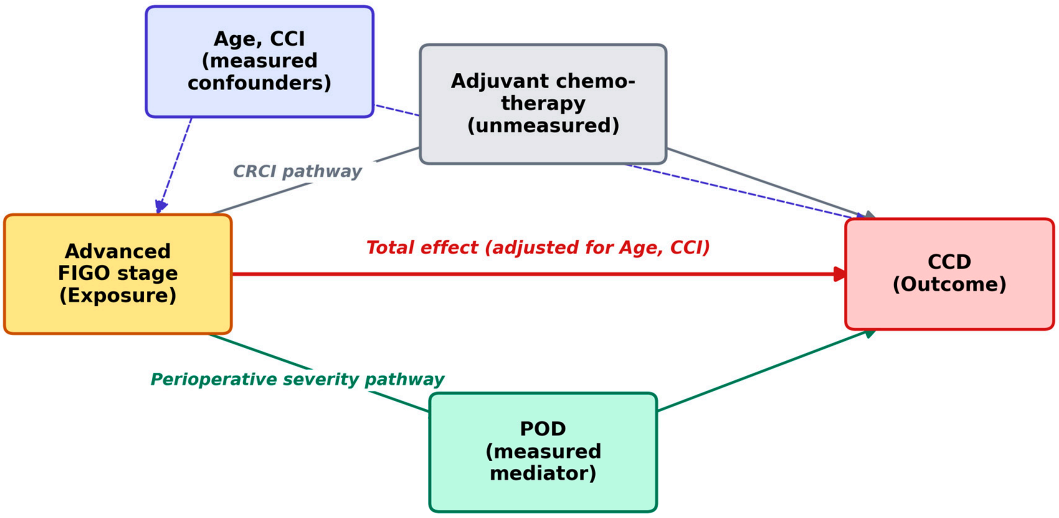

Note: POD is treated as a mediator on the stage→CCD pathway and is therefore not included in the core Fine–Gray model.

Adjuvant chemotherapy (dashed pathway) could not be systematically captured and represents an unadjusted potential mediator.

Appendix S1: R Code for Supplementary Analyses

The following R code generates Figures S1–S2 and Table S7 from the patient-level dataset. Table S4 (visit-frequency adjusted model and follow-up density) is generated by an analogous script appending outpatient visit counts as a covariate. Requirements: R ≥4.3.2 with packages rms, riskRegression, prodlim, cmprsk, survival, and ggplot2.

```
## ========================================
## R Code: Supplementary Analyses
## ========================================

library(rms); library(riskRegression); library(prodlim)
library(cmprsk); library(survival); library(ggplot2)

## --- Data preparation ---
## df: patient-level dataset with columns:
##   time_days: follow-up time in days
##   status: 0=censored, 1=CCD, 2=competing event
##   age, stage (0=I-II, 1=III-IV), CCI

dd <- datadist(df); options(datadist='dd')
kn <- quantile(df$age, probs=c(0.05,0.35,0.65,0.95), na.rm=TRUE)

## --- Fine-Gray model with RCS and interaction ---
fg <- FGR(Hist(time_days, status) ~ rcs(age, kn)*stage + CCI,
          data=df, cause=1)

## --- Figure S1: Interaction plot ---
age_grid <- seq(40, 90, by=1)
med_cci  <- median(df$CCI, na.rm=TRUE)
nd0 <- data.frame(age=age_grid, stage=0, CCI=med_cci)
nd1 <- data.frame(age=age_grid, stage=1, CCI=med_cci)
r0 <- predictRisk(fg, newdata=nd0, times=365)
r1 <- predictRisk(fg, newdata=nd1, times=365)

plot_df <- rbind(
  data.frame(age=age_grid, stage='I-II',  risk=r0),
  data.frame(age=age_grid, stage='III-IV', risk=r1))

ggplot(plot_df, aes(x=age, y=risk*100, color=stage, fill=stage)) +
  geom_line(linewidth=1.2) +
  labs(x='Age (years)',
       y='Predicted 12-month CIF of CCD (%)',
       color='FIGO Stage', fill='FIGO Stage') +
  scale_y_continuous(limits=c(0, 35)) +
  theme_classic(base_size=14) +
  theme(legend.position=c(0.2, 0.85))

## --- Table S7: Stage-stratified CIF at 365 days ---
cif <- prodlim(Hist(time_days, status) ~ stage, data=df)
s <- summary(cif, times=365, cause=1)
## Extract CIF and SE for each stage group
```

```
## RD = CIF(stage=1) - CIF(stage=0)
## Bootstrap 95% CI for RD:
set.seed(42)
boot_rd <- replicate(1000, {
  idx <- sample(nrow(df), replace=TRUE)
  bdf <- df[idx,]
  bc <- prodlim(Hist(time_days, status) ~ stage, data=bdf)
  bs <- summary(bc, times=365, cause=1)
  bs$table[2,'cuminc'] - bs$table[1,'cuminc']
})
quantile(boot_rd, c(0.025, 0.975))

## --- Figure S2: Calibration with optimism correction ---
set.seed(1)
cal <- Score(list(FG=fg),
             Hist(time_days, status) ~ 1,
             data=df, times=365,
             plots='cal', B=500, split.method='bootcv')
plotCalibration(cal, times=365,
                xlab='Predicted 12-month CCD risk',
                ylab='Observed CCD risk')
```

STROBE Statement—Checklist of items that should be included in reports of **cohort studies**

	Item No	Recommendation	Page No
**Title and abstract**	1	(a) Indicate the study's design with a commonly used term in the title or the abstract	1, 3
		(b) Provide in the abstract an informative and balanced summary of what was done and what was found	1, 3
**Introduction**			
Background/rationale	2	Explain the scientific background and rationale for the investigation being reported	4-5
Objectives	3	State specific objectives, including any prespecified hypotheses	6
**Methods**			
Study design	4	Present key elements of study design early in the paper	6
Setting	5	Describe the setting, locations, and relevant dates, including periods of recruitment, exposure, follow-up, and data collection	6
Participants	6	(a) Give the eligibility criteria, and the sources and methods of selection of participants. Describe methods of follow-up	6-7
		(b) For matched studies, give matching criteria and number of exposed and unexposed	6-7
Variables	7	Clearly define all outcomes, exposures, predictors, potential confounders, and effect modifiers. Give diagnostic criteria, if applicable	7-8
Data sources/ measurement	8*	For each variable of interest, give sources of data and details of methods of assessment (measurement). Describe comparability of assessment methods if there is more than one group	7-8
Bias	9	Describe any efforts to address potential sources of bias	8-9; 15
Study size	10	Explain how the study size was arrived at	7-9; 9
Quantitative variables	11	Explain how quantitative variables were handled in the analyses. If applicable, describe which groupings were chosen and why	8
Statistical methods	12	(a) Describe all statistical methods, including those used to control for confounding	8-9
		(b) Describe any methods used to examine subgroups and interactions	8-9
		(c) Explain how missing data were addressed	8-9
		(d) If applicable, explain how loss to follow-up was addressed	8-9
		(e) Describe any sensitivity analyses	8-9
**Results**			
Participants	13*	(a) Report numbers of individuals at each stage of study—eg numbers potentially eligible, examined for eligibility, confirmed eligible, included in the study, completing follow-up, and analysed	9, 26

		(b) Give reasons for non-participation at each stage	9, 26
		(c) Consider use of a flow diagram	9, 26
Descriptive data	14*	(a) Give characteristics of study participants (eg demographic, clinical, social) and information on exposures and potential confounders	9; 21
		(b) Indicate number of participants with missing data for each variable of interest	9; 21
		(c) Summarise follow-up time (eg, average and total amount)	9; 21
Outcome data	15*	Report numbers of outcome events or summary measures over time	9-10; 22
Main results	16	(a) Give unadjusted estimates and, if applicable, confounder-adjusted estimates and their precision (eg, 95% confidence interval). Make clear which confounders were adjusted for and why they were included	10; 23-25
		(b) Report category boundaries when continuous variables were categorized	10; 23-25
		(c) If relevant, consider translating estimates of relative risk into absolute risk for a meaningful time period	10; 23-25
Other analyses	17	Report other analyses done—eg analyses of subgroups and interactions, and sensitivity analyses	10-11; 15
**Discussion**			
Key results	18	Summarise key results with reference to study objectives	11-12
Limitations	19	Discuss limitations of the study, taking into account sources of potential bias or imprecision. Discuss both direction and magnitude of any potential bias	15
Interpretation	20	Give a cautious overall interpretation of results considering objectives, limitations, multiplicity of analyses, results from similar studies, and other relevant evidence	11-16
Generalisability	21	Discuss the generalisability (external validity) of the study results	15
**Other information**			
Funding	22	Give the source of funding and the role of the funders for the present study and, if applicable, for the original study on which the present article is based	17

*Give information separately for exposed and unexposed groups.

Note: An Explanation and Elaboration article discusses each checklist item and gives methodological background and published examples of transparent reporting. The STROBE checklist is best used in conjunction with this article (freely available on the Web sites of PLoS Medicine at http://www.plosmedicine.org/, Annals of Internal Medicine at http://www.annals.org/, and Epidemiology at http://www.epidem.com/). Information on the STROBE Initiative is available at http://www.strobe-statement.org.
